# Variations of the Color of Venous Blood

**Published:** 1858-11

**Authors:** 


*Variations of the Color of Venous Blood.* By Prof. CLAUDE BERNARD.

From the period of the discovery of the circulation, two descriptions of blood have been recognized; the one red or arterial, the other black or venous; and so characteristic has this difference in color of the arterial and venous blood been considered, that it has served since the time of Bichat for the anatomical division of the circulatory organs. The facts now about to be mentioned will prove that henceforth we cannot regard as synonyms the terms venous and black blood; some venous blood being found, in the normal condition, as red as arterial, and even sometimes red and sometimes black. But what will most interest the physiologist is to learn that these variations in the color of venous blood correspond to different determinate functional conditions of the organs.

As long since as 1845, M. Bernard, while experimenting upon dogs on the eliminatory power of the kidney, was surprised at finding that the blood which left that organ by the vein was as red as that which entered it by the artery. Latterly, he has resumed the investigation of the subject at the College of France. The same phenomenon that had formerly been observed in the dog presented itself in the rabbit, and the red blood of the renal veins arriving and visibly mingling with the black blood of the vena cava inferior. The lumbar veins, on the other hand, discharged black blood very near the renal veins, as did also a small muscular vein which opened into the left renal vein. On multiplying the experiments, and varying the condition of the observation, it was soon found that the usual red color of the blood of the renal vein might change in shade, and under the influence of certain circumstances, become completely black. Thus, contradiction would take place if we we were contented with the announcement of a single observation. This may unfortunately be almost always the case in physiology, when we fail to distinguish amidst such complex phenomena, the eminently variable conditions which every living organism presents.

Having exhibited the two possible appearances of the blood of the renal veins, the next point was to examine what relation they held to the performance of the function of the kidney. To this end there was placed in the ureter a small silver tube, by which the urine was observed to flow drop by drop, almost continuously, as it is known to do. It was then found that the blood of the renal vein, as well as the tissue of the kidney, continued completely red as long as the urine flowed abundantly by the tube; but that this flow ceased to take place under the influence of circumstances which, while they rendered the blood of the renal vein black, gave at the same time a bluish tint to the kidney itself. Hence it would seem that the red color of the blood of the renal vein was referable to the functional activity of the kidney, and its black color to the condition of repose or cessation of function. Moreover, it was found that the reaction of the urine excited no influence on the phenomenon; the blood of the renal vein being alike red in the dog with acid urine and in the rabbit whose urine is alkaline during digestion and acid after twenty-four or thirty-six hours of abstinence.

Without enumerating the various influences that are capable of disturbing the secretion of the urine, and leading to a change in the color of the blood of the renal vein, the author contents himself with adverting to the perturbation that may ensue from the mode of operating. If it be desired to observe the red color of the venous blood, the abdomen must not be largely opened and the intestines displaced, for then suppression of urine will almost immediately ensue, and the blood in the vein will often become as black as that of the vena cava. A small opening should be made in the lumbar region, and especially on the left side, the left renal vein being more easily exposed in consequence of its greater length. Through the same wound the ureter may afterwards be isolated, so as to introduce the silver tube, in order to make certain whether during the observation the function of the kidney continues to be carried on or not.

From what has been said it clearly results that the blood of this vein, presenting habitually a red color in connection with the formation of urine, which is almost continuous, does not correspond with the definition of venous blood already given. The first question that presents itself to the mind is extended to the secretory organs, whose functions is, in like manner, the separation of a special organic fluid in their tissue. The submaxillary gland of the dog offers great facilities by its superficial and isolated position for the investigation of this point; the numerous anatomical varieties which its vein exhibits in nowise interfering with the observation of the physiological phenomena. In the first experiment, the blood it contained was found as completely black as the darkest venous blood; but this arose from the fact of the secretion by the gland, which is intermittent, not then being active. When, however, some drops of vinegar were instilled into the mouth of the animal, which by its reflex action induced the secretory activity of the gland, the blood gradually but rapidly exchanged the black for the red color, the black color being again restored when the secretion had ceased. In order to render the interpretation of the phenomenon yet more positive, a small silver tube was introduced into the exposed execretory canal of the gland, and the nervous Branch of the lingual nerve going to the gland was isolated. It was then found that as long as the organ remained in repose nothing passed by the tube, and black blood passed through the vein; while, whenever the nerve of the gland was excited by galvanism, and secretion was produced, the color of the blood became red, regaining its black color when the secretion was arrested after the cessation of the stimulation. The trial was made several times and with similar results. There was always an interval of some seconds elapsed between the production of the cessation of the stimulus and the changes of color in the blood, as if the gland required a short period for the discharge of the blood it already contained. Moreover, it was observed that the blood flowed always more abundantly when red, that is, during the state of functional activity of the organ, than when black, the organs being then in a state of repose.

These two series of results obtained from the kidney and the submaxillary gland certainly do not constitute isolated facts; and the same observation should doubtless be extended to other glands. The investigations as yet made by the author on the parotid and on the glands of the alimentary canal have furnished similar general results; but the study of the subject will only be complete when each particular gland shall have been experimented on.

" It results from the facts now stated, that in the physiological condition we may still designate arterial blood as the red blood, the appellation of black blood cannot in this general manner be conferred on venous blood. We have seen, in fact, that the venous blood may be found red or black in secretory organs, accordingly as these are observed in a state of activity or repose. This consideration of the activity and repose of the organ, which correspond in some degree to its statical and dynamical conditions, appears to me to be an important point for introduction into the chemical and physiological study of the blood. In fact, it is not alone by its color that the venous blood of an organ at rest differs from the venous blood of an organ in activity; but it presents also other important differential characters, which must depend upon a profound difference of the chemical constitution. Thus the venous blood of the kidney performing its function, which is red, is more diffluent, and sometimes even presents no coagulum; while the blood of the same vein, when the kidney has ceased to act, becomes black, and presents a consistent coagulum, etc. Without doubt physiologists and chemists have already learned that venous blood cannot, like arterial blood, be everywhere regarded as identical, and that it is necessary to analyze the venous blood of each organ in particular; but what, I believe, has never yet been stated, and which, nevertheless, seems to me indispensably necessary to be henceforth considered, if we desire that chemical analyzes should prove as useful as possible to physiology, is the separate and comparative examination of the composition of the venous blood of the same organ when in repose and in activity. From what has been stated above, we may predict that there will be often found greater differences between the two bloods of the same organ in the state of activity and of repose than between the corresponding bloods of two different organs. This point of view is not solely applicable to glands, but should be extended to all the organs of the body, the venous blood of which will not have to be studied under these two conditions. We may in some measure characterize each tissue by the very different modifications its functional activity impresses on the blood that traverses it. Thus, if the blood quits glands when active reddened, it leaves a muscle that contracts, with a very black color, and different physical characters. The mechanism of these different colorations of the blood will necessarily receive its explanation through ulterior chemical analyses, of which we are only at present desirous of indicating the physiological conditions. We will conclude with the remark, that all these modifications which supervene in the blood as a consequence of the functional activity of organs are always determined by the nervous system. It is consequently at this point of contest between the organic tissues and the blood that we must acquire our ideas of the special part played by the nervous system in the physico-chemical phenomena of life. The development of the facts which relate to this point of general physiology will form the subject of an early communication."—*Med. Times and Gaz.*, Feb. 27, 1858, from *Comptes Rendus*, tom. xivi. pp. 159—165.

---

*On the Properties and Uses of the Red Blood and of the Dark Blood.*—Dr. Brown-Sequard communicates several observations which strengthen him in the view that blood charged with oxygen, whether arterial or venous, has the power of reëstablishing the vital properties of the contractile and nervous tissues, when äpplied within a certain space of time, after they have lost these properties. After having repeated Sir Astley Cooper's experiments, that animals die from asphyxia, when circulation is stopped in the four encephalic arteries, and that they recover almost immediately if the circulation is quickly re-established—Brown Séquard ascertained that if the circulation takes place again a few minutes after the last respiratory movements had ceased, life does not re-appear. But the red blood then still possesses the power of re-establishing life, as insufflation of the lungs causes energenic movements of the limbs, while the head remains at rest as long as the pressure on the arteries continues; but as soon as the latter is removed, the encephalon resumes its functions, and the animal may be restored to full life, even fifteen minutes after the commencement of the compression. The same author found, also, that in heads separated from the trunk, injections of red blood—*i. e.*, blood charged with oxygen—may reproduce the actions of the encephalon (respiratory movements of the face, movements of the eyes, &c.) Further experiments have shown, 1. That the presence of fibrin in the blood is not necessary for this effect; 2. That serum alone does not possess this power; 3. That the blood richest in oxygen and globules does possess it in the highest degree—the globules acting probably only as bearers of oxygen. Another series of experiments regarding the properties of red and black blood, leads Brown-Séquard to the inference, that the red blood increases the vital properties by nourishing the tissues, but that it is incapable of making these properties appear by stimulating them; that the black blood is an energetic stimulant of the nervous centres, and also, but in a less degree, of the nerves and contractile tissues, but that it does not possess—or at least only in a small degree—the power of maintaining or regenerating the vital properties.—*B. and F. Med.-Chirurg. Rev.*, Jan. 1858, from *Compt. Rendus*, Oct. 19, 1857.

